# Advance Treatment Provision (DAT): Italian Legislation in the European Context. A Comparative Analysis

**DOI:** 10.3389/fpubh.2022.826194

**Published:** 2022-04-06

**Authors:** Maria Silvestre, Serena Corradi, Benedetta Pia De Luca, Alessandra Stellacci, Alessandro Dell'Erba, Maricla Marrone

**Affiliations:** Section of Legal Medicine, Interdisciplinary Department of Medicine (DIM), Bari, Italy

**Keywords:** living wills, Italian legislation, European Union, diagnostic tests, therapeutic choices, individual health treatments

## Introduction

The Italian legal system recognizes informed consent, or the consent given by the patient or by the patient following exhaustive information, as a principle linked to the dignity of the person.

Art. 32, paragraph 2, of the Italian Constitution provides that “No one can be obliged to a specific health treatment except by law (...).”

As the Constitutional Court has also confirmed, the consensus is configured today as it is “True personal right and is based on the principles expressed in art. 2 of the Constitution, which protects and promotes their fundamental rights, and in arts. 13 and 32 of the Constitution, which establish, respectively, that “personal freedom is inviolable,” and that “no one can be obliged to a specific medical treatment except by law” “(Constitutional Court, sentence no. December 23, 2008).”

The constitutional framework was then recently supplemented by law no. 219/2017, approved by the Italian Parliament, according to which “no health treatment can be started or continued without the free and informed consent of the person concerned, except in cases expressly provided for by law.” (art. 1, c. 1). On 31.1.2018, Law n. 219 of 22.12.2017 (1), containing “Rules on informed consent and advance treatment provisions,” better known as the “law on living wills” has been approved. The law is made up of eight articles, of which the first five introduce significant innovations, dealing with the following aspects: the right of each patient to know their health condition, consenting, or refusing health treatments with full knowledge; the doctor's duty to work to alleviate the patient's suffering, without this becoming useless therapeutic fury; the principle of the full lawfulness of refusing treatment through the right of each subject to express, in anticipation of an inability to self-determine, their binding preferences; the right of the minor and the incapable of receiving information on their situation and expressing their will, while remaining the consent or dissent of competence of the person exercising parental responsibility or of the guardian; the right to care planning between doctor and patient. The law focuses on palliative care and pain therapy, such as care and assistance aimed at examining the patient's physical and mental suffering, in order to improve their quality of life, while respecting their dignity. They received discipline in Italy with l. 38/2010 (2). In 2013, the WHO unanimously approved a document that commits all states to developing palliative care and indicates it as a fundamental human right (3). In particular, sedation is a therapeutic act that aims to relieve the patient's stress and suffering by controlling refractory symptoms. The new law, however, provides, in art. 2, that the doctor, even taking into account the will of the patient (if it has been expressed), must refrain from obstinacy in therapeutic and/or diagnostic treatments from which no benefit for the health of the sick person or an improvement can be expected for the quality of life of the same. In order to speak of “therapeutic persistence,” there must be, at the same time: the documented uselessness and ineffectiveness of the therapies, the severity of the treatment, and the exceptional nature of the therapeutic means. The patient, in turn, cannot expect health treatments that are contrary to legal regulations, professional ethics, and good clinical-care practices: if such requests are made, the doctor has no professional obligation. A major novelty is the provision in the law of the possibility, for every adult capable of understanding and willing, to express, through Advance Treatment Provisions (DAT), their wishes regarding health treatments, as well as their consent or refusal. To diagnostic tests, therapeutic choices, individual treatments “in anticipation of a possible future inability to self-determine and after having acquired adequate medical information on the consequences of his choices.” This is the first complete codification of the provisions of will in health matters, so far governed only by private autonomy.

## Living Will: Italian Features

Advance treatment provisions, commonly referred to as “living will” or “bio testament,” are regulated by art. 4 of Law 219 of 22 December 2017, which entered into force on 31 January 2018.

“Every person of age and capable of understanding and willing, in anticipation of a possible future inability to self-determine [...], can, through the Advance Treatment Provisions, express their wishes regarding health treatments, as well as consent or the refusal with respect to diagnostic tests or therapeutic choices [...].” In anticipation of a possible future inability to self-determine and after having acquired adequate medical information on the consequences of their choices, the law provides for the possibility for each person to express their wishes regarding health treatments, as well as consent or refusal on:

(1) Diagnostic tests;
(2) Therapeutic choices;
(3) Individual health treatments.

There are no forms required by law, however, some municipalities have prepared facsimile models.

The drafting of the DAT can take place in different forms:

- By the notary (both with public deed and with private deed in which the person writes his will independently and has the signatures authenticated by the notary), in both cases the notary keeps the original;
- At the civil status office of the municipality of residence (with private agreement) which records it in a special register, where established (see the circular of the Ministry of the Interior);
- At the competent health facilities in the regions that have regulated the collection of DATs (with private agreement);
- At the Italian consular offices, for Italian citizens abroad (in the exercise of notary functions).

The DATs are exempt from the obligation of registration, stamp duty and any other tax, duty, right, and tax.

In the event that the physical condition of the patient does not allow it, the DAT can be expressed through video recording or devices that allow the person with disabilities to communicate.

In the same forms, the DATs are renewable, modifiable and revocable at any time. In cases where “reasons of emergency and urgency prevented the revocation of the DAT with the forms provided for in the previous periods, these can be revoked with a verbal declaration collected or videotaped by a doctor, with the assistance of two witnesses.”

Law 219 provides for the possibility of indicating a trustee in the DAT, whose choice is completely left to the will of the settlor. The law limits itself to providing that the trustee is of age and capable of understanding and willing. The trustee is called upon to represent the interested party in relations with the doctor and health facilities.

The doctor is required to comply with the DAT, which can be disregarded, in whole or in part, by the doctor himself, in agreement with the trustee if:

- The DATs appear clearly incongruous or do not correspond to the current clinical condition of the patient;
- There are therapies that cannot be foreseen at the time of subscription, capable of offering concrete possibilities for improving living conditions.

In the event of a conflict between the trustee and the doctor, the decision is left to the tutelary judge.

In the event that the DAT does not contain the indication of the trustee or the trustee has renounced it or is deceased or becomes incapable, the DAT remains effective with regard to the will of the settlor. In case of need, the tutelary judge appoints a support administrator.

The 2018 budget law (law 205/2017, article 1, paragraphs 418 and 419) provided for—for the purposes of implementing the aforementioned discipline—the establishment of a database for the registration of DATs at the Ministry of Health (1).

The regulation approved by decree 10 December 2019, n. 168 regulated the methods for collecting copies of the DATs in the national database, for which art. 1, paragraphs 418 of the aforementioned 2018 budget law authorized the expenditure of 2 million euros for 2018, while the 2019 budget law (Article 1, paragraph 573, of law no. from 2019, the expenditure of 400 thousand euros per year for the financing of the maintenance and management costs of the information system of the database) (4).

The regulation, which allows the implementation of the database, provides in particular for the regulation of its functions, the information contents and the subjects who are authorized to feed it, as well as the methods of accessing the data. A specific rule aims to provide for interoperability between the national database, the unitary network of notaries and the other regional databases provided for by the law in question.

### Objectives

The primary objective of this work is to compare the legislation in force in the 28 countries of the European Union relating to the area of living wills, so that the legislation in each country can be evaluated.

The secondary objective of the work is the analytical and critical evaluation of the similarities and differences between the various European regulations, with a view to achieving a more homogeneous framework.

### Methodology

First of all, law 219/2017 was analyzed with particular reference to article 4 relating to living wills.

A search was also conducted for the laws of the European countries within the EU: Germany, Austria, Belgium, Bulgaria, Cyprus, Croatia, Denmark, Slovakia, Slovenia, Spain, Estonia, Finland, France, Greece, Hungary, Ireland, Italy, Latvia, Lithuania, Luxembourg, Malta, the Netherlands, Poland, Portugal, the United Kingdom, the Czech Republic, Rumania, and Sweden.

Once all of this information had been collected, and following Italian law major features (Table 1), we drew up a comparative table to show the differences among the laws among European countries so that they could be studied (Table 2).

**Table 1:** Italian living wills.

**Normative reference**	**Law 219/2017**
Mandatory	No
Compilation period	In written form or through video recordings or, for the person with disabilities, through devices than allow them to communicate
Formalization	Yes: they must be drafted by public deed or by authenticated private writing or by private writing delivered personally by the device at the office of the civil status of the municipality of residence of the same device, which provides the registration or registration in the healthcare facilities, if the conditions referred to in paragraph 7 of the law
Designation of a representative	Yes
Registry	Yes
Requires revisions	No
Modifiable at any time	Yes
Revocable	Yes

**Table 2:** Characteristics of European Union countries legal system in the field of living wills.

	**Specific regulations**	**Binding**	**Written vs. oral format**	**Formalization**	**Designation of representative**	**Registry**	**Requires revisions**	**Modifiable at any time**	**Revocable**
Italy	Yes (2017)	No	Written or video recording	Yes	Yes	Yes	No	Yes	Yes
Germany	Yes (2009)	Yes	Written	Not necessary	No	No	No	Yes	Yes
Austria	Yes (2006)	Yes	Written/oral	Notary Lawyer A	Yes	No	Yes, every 5 years	Yes	Yes
Belgium	Yes (2002)	Yes	Written	Not necessary	Yes	No	No	Yes	Yes
Denmark	Yes (2005)	No	Written	Registry Clinical history	No	Yes	No	Yes	Yes
Slovenia	Yes (2008)	Yes	Written	Specific form	Yes	No	Yes, every 5 years	Yes	Yes
Spain	Yes (2002)	Yes	Written	Notary Witness A	Yes	Yes	No	Yes	Yes
Estonia	Yes (2001)	Yes	Written/oral	Not necessary	No	No	No	Yes	??
Finland	Yes (1992)	Yes	Written/oral	Not necessary	Yes	No	No	Yes	Yes
France	Yes (2002)	No	Written	No	No	No	Yes, every 3 years	Yes	Yes
Hungary	Yes (1997)	Yes	Written	Notary	Yes	No	Yes, every 2 years	Yes	Yes
Latvia	Yes (2009)	Yes	Written	Inform health center	Yes	No	No	Yes	Yes
Luxembourg	Yes (2014)	Yes	Written/oral	No	Yes	No	No	Yes	Yes
The Netherlands	Yes (1995)	Yes	Written	Not necessary	Yes	No	No	Yes	Yes
Portugal	Yes (2012)	Yes	Written	Registry notary	Yes	Yes	Yes, every 5 years	Yes	Yes
The United Kingdom	Yes	Yes	Written/oral	Witness	Yes	No	No	Yes	Yes

### Italy vs. European Scenary

We express the results of the analysis by specifying each section of the comparative table described above (Table 2):

### The Existence of a Specific Normative Reference on Living Wills

Of the 28 countries of the EU, only 16 have specific law covering LWs. Of these countries, the majority have ratified the Oviedo Convention. Almost all of the countries with specific laws make them legally binding, except for Denmark, Italy and France, in which the law is a guideline for decision making.

#### Compilation Method

In general written expression is selected as the valid form, except in Finland and Estonia, where an oral expression is also valid. In Italy, in case the physical condition of the patient does not allow it, living wills can be expressed through video recording or devices that allow the person with disabilities to communicate.

#### Formalization

It depends on the country. It may take place before a notary, before several witnesses or before the Administration, or it may not have a specific form. In Italy, the DATs must be drawn up by public deed or by writing private authenticated or by private writing delivered personally by the settlor at the civil status office of municipality of residence of the settlor himself, who provides the entry in the appropriate register.

#### Designation of a Representative

The designation of a representative in the field of health is considered in 12 countries (Italy is included) and this may be specified in the living will document or in another document, depending on the country. According to Italian law, the trustee must be an adult and capable of to understand and to want: in the event that the DAT does not contain the indication of trustee or he has renounced it or has died or has become incapable, the DAT remain effective with regard to the will of the settlor. Four countries (Germany, France, Denmark, and Estonia) do not consider the designation of a legal representative.

#### Registration

In Italy, the 2018 Budget Law in paragraphs 418 and 419 of article 1 provided for and financed the establishment at the Ministry of Health of a database for the registration of advance treatment provisions (DAT) through which every adult and capable to understand and want, in anticipation of a possible future inability to self-determine, can express their wishes regarding individual health treatments, as well as consent or refusal with respect to diagnostic tests or therapeutic choices and individual health treatments. The Decree n. 168 of 10 December 2019, published in the Official Journal no. 13 of January 17, 2020, governs the procedures for registering DATs in the national database.The Italian DAT database has the function of:

- collect a copy of the advance processing provisions;
- ensure timely updating in the event of renewal, modification or revocation;
- ensure full accessibility of the DATs both by the doctor who is treating the patient, in situations of inability to self-determine, both by the settlor and by the trustee possibly appointed by him.

The database also records a copy of the appointment of any trustee and the acceptance or renunciation of these or the subsequent revocation by the settlor.

Only four countries have a defined registry (Denmark, Spain, Italy, and Portugal). In the other European countries the living will is generally present in the patient's clinical history.

#### Regular Revisions

In five European countries the regular revision of a living wills is indicated, without time indications. In other countries it is strongly suggested that the expressed will is as recent as possible, so as to be current with respect to the clinical condition.

#### Modifications

In every european country any living wills modification is allowed without time limits.

#### Revocation of Living Wills

It is possible to revoke a LW in all the countries, either in written form or oral form, although written form is strongly suggested.

## Conclusions

The signing of the Oviedo Convention stimulated interest in LW across Europe. However, cultural, legislative and religious factors have led to clear differences between EU countries. Although the Oviedo Convention is one of the most important human rights documents in the world, not all EU countries have yet adopted it. Not having ratified this convention does not mean that the countries in question do not have laws governing this field. The important thing is that the guidelines of the Oviedo Convention must be applicable in any country that has ratified it. However, there is still no agreement between the different countries on how to understand and accept the scope of the document (1).

The binding nature of the LWD means that it has legal validity and must therefore be respected. The way in which it is considered binding varies from country to country. In France, like in Italy, it is not considered binding (2, 3); in Austria it must be signed in front of a notary, whereas in the United Kingdom the importance of the choice made by the medical team arises when there is a clear and well-justified justification for this (4); in Germany and the Netherlands several conditions must be met in order to qualify as binders (5, 6).

If the format of the LWD is written or oral, most countries, including Italy, opt for a written format. In Italy, in case the physical condition of the patient does not allow it, the LW can be expressed through video recordings or devices that allow the person with disabilities to communicate. Only Finland and Latvia accept an oral expression of will, although their Laws leave doubts about the validity of this format. The formalization of LWD is an administrative procedure that changes from one country to another. In Italy, the drafting of the LWD can be done by the notary (either by public or private act in which the person writes his will independently and has the signatures authenticated by the notary), in both cases the notary retains the original; at the civil status office of the municipality of residence (with private agreement) which registers it in a special register, where established (see circular of the Ministry of the Interior); at the competent health facilities of the regions that have regulated the collection of LWD (subject to private agreement); at the Italian consular offices, for Italian citizens abroad (in the exercise of notarial functions). The formalization in Austria (6) requires the evaluation of experts of the person issuing the LWD, which must then be presented in front of a notary, lawyer or representative of the Administration, and must also be paid taxes.

In Spain it is required the signature of a notary or that of three witnesses without family or contractual relationships. In Hungary (7), the process requires assessment by a doctor, a specialist in the field and a psychiatrist. Other countries do not require any specific procedure is the patient and his family members or those close to him to inform the LW doctor. Another interesting option would be to include LWD as part of the clinical history allowing the patient to make decisions about his future after receiving information about his health, treatment options and diagnostic procedures (8). The appointment of a representative is one of the key points, his powers vary from one country to another. Some legal systems opt for the creation of the representative, with more or less opportunities for family members to offer informal representation, and of course with the possibility of giving the medical team the power to make certain decisions at certain times. In Spain, Portugal, Luxembourg, and the Netherlands the representative acts as an interlocutor with the medical team to interpret the LW and ensure that it is taken into account in the decision-making process (9). This form of representation is limited to the field of health and disease and works only when the patient is unable to express his wishes. In Italy the Law 219 provides for the possibility of indicating a trustee in the LWD, whose choice is completely left to the will of the settlor. The law limits itself to providing that the trustee is of age and capable of understanding and willing.

The trustee is called upon to represent the interested party in relations with the doctor and health facilities.

Normally no other document is needed to formalize such representation and the representative is usually appointed in the same LWD, thus speeding up the procedure. In Denmark, Germany, Estonia, and France the role of the representative is not limited to the field of health, as it can facilitate decision-making in all aspects of life.

In the United Kingdom, the appointment of a representative involves a specific procedure and generates a new document. The Office of the Public Representative shall also be notified by means of a document setting out the limits to the decisions which the representative may take. They shall also determine the considerations which the representative must take into account when taking decisions, seeking the best interests of the represented person and thus supporting the alternative judgment (10). Belgium, Slovenia, Finland, and Hungary require the formalization of a parallel document for an individual to be a representative. Informal representation by family members is possible in the absence of such a document (11). Austria allows representation by family members or trusted persons, provided that they are entered in a specific register (12).

In some EU countries (Spain, Portugal, and Denmark) a LW Register has been introduced to facilitate access by medical staff to care for a person in any geographical location (13). In addition to using this Register, Medical personnel may also receive a patient's LW if someone or the patient itself provides a copy of the same properly completed and validated. The existence of the Registry has another possible benefit associated with the privacy of the process, as some people prefer to run this process in a more confidential manner. Access to this information is granted to the petitioner, his representative and the medical team. In Italy, the Budget Law of 2018 in paragraphs 418 and 419 of Article 1 provided for and financed the establishment at the Ministry of Health of a database for the registration of advance treatment orders (DAT). LWD can change over time, depending on the patient's clinical circumstances. A regular review of the LWD would be desirable to ensure that it expresses the wishes of those who drafted it according to its condition. In France and Hungary a deadline is set for the validity of the document, with the need to renew them every 2–3 years, in Italy there is no period of validity of the same.

With regard to changes to the content of a LWD, all countries provide that they are renewable, modifiable and revocable at all times. The possibility of withdrawal would prevent the possibility that obsolete and unwanted LW is not applied when the patient has changed his mind. In conclusion, our review has shown that just over half of EU countries have developed specific legislation in this field, partly in view of the heterogeneous nature of the legislation which makes it difficult to implement. Of the 13 countries that have yet to pass their laws, the Czech Republic, Bulgaria, Croatia, Greece, Cyprus, Slovakia, Lithuania, and Romania have ratified the Oviedo Convention. In Italy, the 2019 law of 22 December 2017 regulates, among other things, the anticipated provisions of treatment, that is, the free expression of will expressed by the patient or in any case by the person with regard to their decisions regarding information on their state of health, on diagnostic examinations and medical treatment, taking account of the provisions of the Oviedo Convention.

The hope is that sooner or later we can find a uniformity in the discipline that regulates the expression of the patient's will to treatment, so as not to create differences neither in the rights nor in the duties of the various figures involved in the health path.

## Data Availability Statement

The original contributions presented in the study are included in the article/supplementary material, further inquiries can be directed to the corresponding author/s.

## Author Contributions

All authors listed have made a substantial, direct, and intellectual contribution to the work and approved it for publication.

## Conflict of Interest

The authors declare that the research was conducted in the absence of any commercial or financial relationships that could be construed as a potential conflict of interest.

## Publisher's Note

All claims expressed in this article are solely those of the authors and do not necessarily represent those of their affiliated organizations, or those of the publisher, the editors and the reviewers. Any product that may be evaluated in this article, or claim that may be made by its manufacturer, is not guaranteed or endorsed by the publisher.
